# SPRi Biosensor for Simultaneous Determination of HIF-1α, Angiopoietin-2, and Interleukin-1β in Blood Plasma

**DOI:** 10.3390/s24175481

**Published:** 2024-08-24

**Authors:** Zuzanna Zielinska, Lukasz Oldak, Tomasz Guszcz, Adam Hermanowicz, Ewa Gorodkiewicz

**Affiliations:** 1 Bioanalysis Laboratory, Doctoral School of Exact and Natural Science, Faculty of Chemistry, University of Bialystok, Ciolkowskiego 1K, 15-245 Bialystok, Poland; z.zielinska@uwb.edu.pl; 2 Bioanalysis Laboratory, Faculty of Chemistry, University of Bialystok, Ciolkowskiego 1K, 15-245 Bialystok, Poland; l.oldak@uwb.edu.pl (L.O.); ewka@uwb.edu.pl (E.G.); 3 Department of Urology, Hospital of Ministry of Interior and Administration in Bialystok, Fabryczna 27, 15-471 Bialystok, Poland; tomasz.guszcz@o2.pl; 4 Pediatric Surgery Department, Medical University of Bialystok, 15-089 Bialystok, Poland

**Keywords:** biosensor, angiogenesis, surface plasmon resonance

## Abstract

A new analytical method, based on SPRi biosensors, has been developed for the simultaneous determination of the pro-angiogenic factors HIF-1α, angiopoietin-2 (ANG-2), and interleukin-1β (IL-1β) in biological fluids. These proteins take part in the process of angiogenesis, i.e., the creation of new blood vessels, which is a key stage of cancer development and metastasis. A separate validation process was carried out for each individual compound, indicating that the method can also be used to study one selected protein. Low values of the limit of detection (LOD) and quantification (LOQ) indicate that the developed method enables the determination of very low concentrations, in the order of pg/mL. The LOD values obtained for HIF-1α, ANG-2, and IL-1β were 0.09, 0.01, and 0.01 pg/mL, respectively. The LOQ values were 0.27, 0.039, and 0.02 pg/mL, and the response ranges of the biosensor were 5.00–100.00, 1.00–20.00, and 1.00–15.00 pg/mL. Moreover, determining the appropriate validation parameters confirmed that the design offers high precision, accuracy, and sensitivity. To prove the usefulness of the biosensor in practice, determinations were made in plasma samples from a control group and from a study group consisting of patients with diagnosed bladder cancer. The preliminary results obtained indicate that this biosensor can be used for broader analyses of bladder cancer. Each of the potential biomarkers, HIF-1α, ANG-2, and IL-1β, produced higher concentrations in the study group than in the control group. These are preliminary studies that serve to develop hypotheses, and their confirmation requires the analysis of a larger number of samples. However, the constructed biosensor is characterized by its ease and speed of measurement, and the method does not require special preparation of samples. SPRi biosensors can be used as a sensitive and highly selective method for determining potential blood biomarkers, which in the future may become part of the routine diagnosis of cancers.

## 1. Introduction

Angiogenesis is the process of creating new blood vessels from existing ones. In the body’s normal physiological state, it is associated with wound healing, embryogenesis, and ovulation. In pathological processes it is associated with, among others, diabetic retinopathy, cardiac ischemia, rheumatoid arthritis and, most importantly, tumor growth and metastasis [1,2]. The growth, invasion, and migration of endothelial cells are the basic stages of the angiogenesis process and are regulated by a precise system of pro- and anti-angiogenic factors [3]. Cancer angiogenesis takes place through “germination”, namely the formation of new branches, but also by increasing the diameter, elongation, and division of already existing vessels [4]. The emerging vessels of cancerous tumors differ significantly from those that are properly formed. Physiologically occurring blood vessels have a stable structure, ensure rapid blood flow, and do not form so-called capillaries, i.e., small, delicate, and twisted vessels, as is the case with tumor angiogenesis. They have numerous protuberances and may be irregular, tangled, and thickened. Blood flow in such vessels is also slower. Much data indicate that the vessels formed during cancer progression do not perform basic physiological functions and do not provide tumors with sufficient nutrients and oxygen [5].

**Figure 1:**
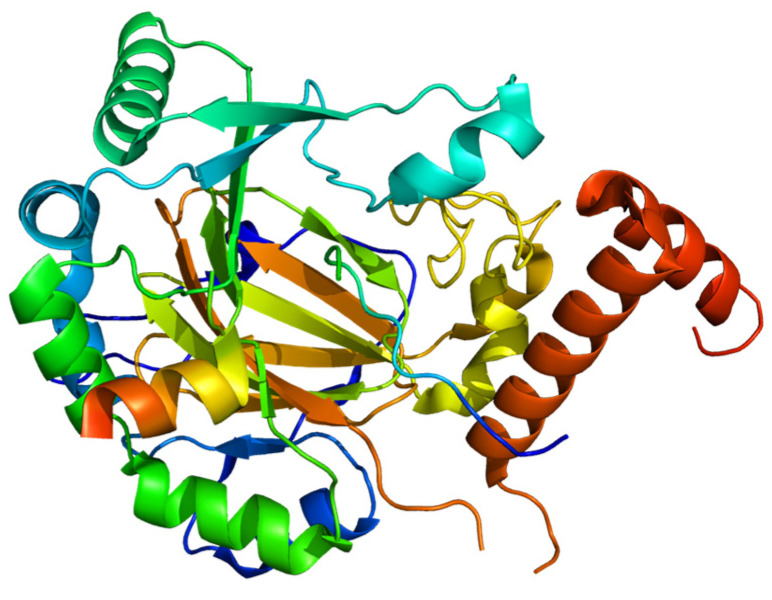
HIF-1α protein structure [9].

Hypoxia is a state always present in the tumor environment due to the rapid growth of diseased cells which is not accompanied by an adequate physiological blood supply, and therefore the availability of oxygen and nutrients is limited. In response to the state of hypoxia in the tumor environment, factors stimulating angiogenesis produced by cancer cells induce the formation of a new blood supply to the tumor, namely the creation of a new blood vessel, which affects its further growth and development. One such angiogenic factor is HIF-1α, which maintains appropriate conditions for tumor development through metabolic adaptation to low oxygen levels and the creation of blood vessels, which favor the survival of cancer cells [6]. HIF-1α is one of the isoforms of the 92 kDa HIF protein, and plays a part in carcinogenesis and tumor growth by regulating genes involved in angiogenesis, metabolism, and other biological mechanisms. Its presence in the tumor environment may promote, among other things, the expression of VEGF (vascular endothelial growth factor), another protein involved in vessel formation; factors inducing the degradation of the extracellular matrix; and the TWIST gene, which is involved in the mechanisms of metastasis. Increased HIF-1α expression can be observed in many cancers, such as stomach, bladder, colon, pancreas, kidney, ovary, and brain cancer [7,8]. The structure of HIF-1α is shown in Figure 1 [9].

**Figure 2:**
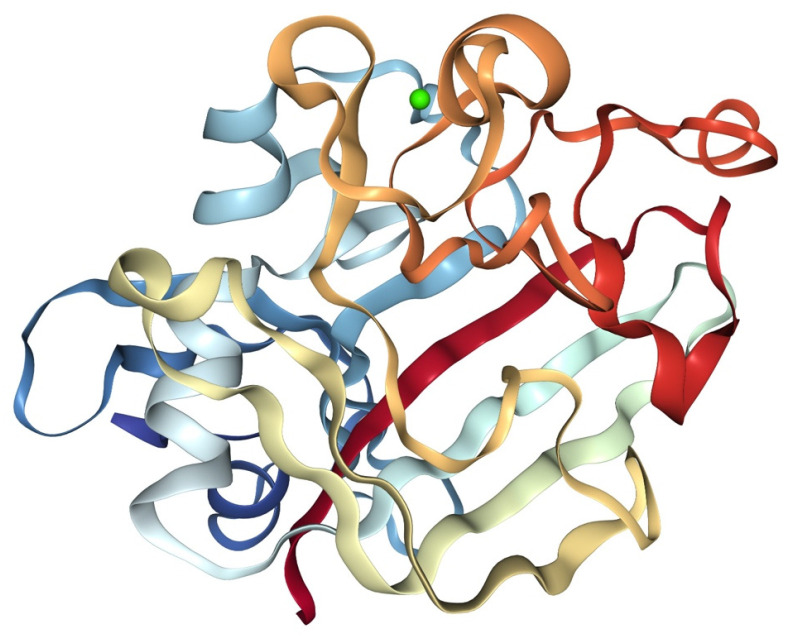
ANG-2 protein structure [18].

Angiopoietin-2 (ANG-2) is a growth factor with a mass of approximately 70 kDa, which belongs to a four-member family of proteins (ANG-1, ANG-2, ANG-3, and ANG-4) with the two related receptors Tie2 and Tie1 [10]. These receptors, more commonly called tyrosine kinase receptors, can be found on vascular endothelial cells, and also occur in the presence of certain macrophages involved in the process of vessel formation [10]. Regarding angiopoietins, the most studied of the group are angiopoietin-1 and angiopoietin-2. ANG-1 is thought to play a role in vascular maturation, as well as in the migration, adhesion, and survival of circulating endothelial cells [11]. ANG-2 is located precisely in these cells, in places where vascular remodeling is to take place. Angiopoietin-1 stabilizes blood vessels by increasing interactions in endothelial cells and the extracellular matrix. Angiopoietin-2 acts antagonistically to ANG-1—it competitively binds to Tie2, which destabilizes vessels [12]. Interestingly, the pro-angiogenic effect, and therefore the stabilization of vessels, is dependent on the presence of HIF-1α and, most importantly, the activity of VEGF. In the absence of VEGF, angiopoietin-2 affects vascular regression due to induction of endothelial cell apoptosis. However, when VEGF is highly active, it acts as a pro-angiogenic factor [13]. Studies indicate that the dynamic balance between ANG-1 and ANG-2, as well as VEGF activity, control tumor angiogenesis. In normal, physiologically functioning tissue, ANG-1 activity predominates, while higher ANG-2 expression is observed in cancer cells [12]. Increased levels of this protein can be observed in, for example, colorectal cancer [14], lung cancer [12], liver cancer [15], glioma and ganglioblastoma [16], and also bladder cancer [17]. In the latter case, ANG-2 expression is related to the stage of disease advancement—in studies, it has indicated poor prognosis and patient survival. The structure of ANG-2 is shown in Figure 2 [18].

**Figure 3:**
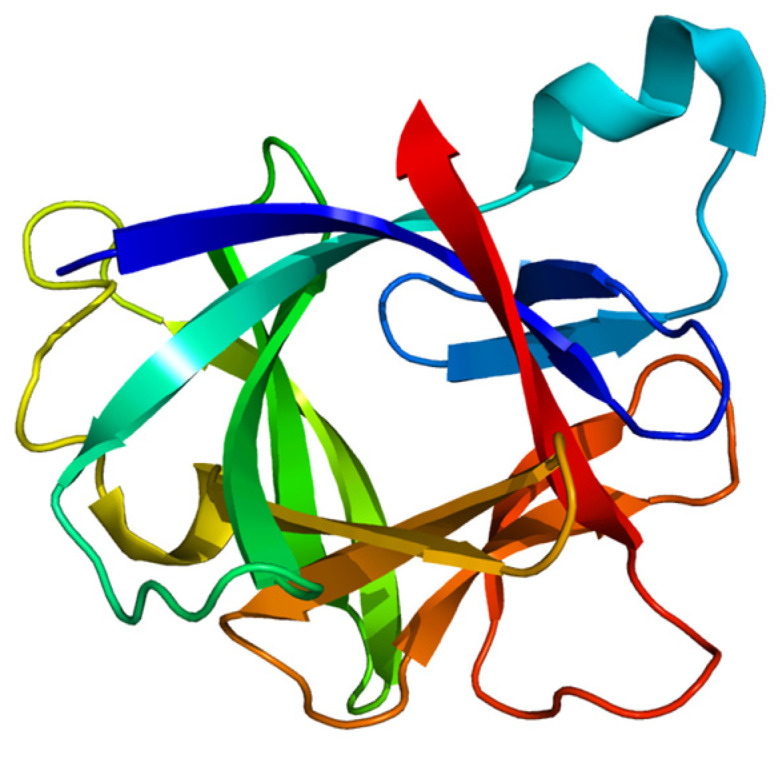
IL-1β protein structure [33].

Cancer angiogenesis is usually accompanied by inflammation, which is not observed during physiologically occurring angiogenesis [19]. Therefore, both processes—angiogenesis and inflammation during cancer development—work together through the activation of pro-inflammatory cells, along with the activation and secretion of pro-angiogenic cells. One of the compounds that characterizes inflammation is interleukin-1β (IL-1β) [20]. The synthesis and processing of this protein are controlled, and require two signals—“priming”, which allows the transcription of the IL-1β gene, and an activation signal, which leads to the activation of appropriate inflammatory complexes and caspases to cleave inactive pro-IL-1β, with a mass of 31 kDa, into the active, mature form of IL-1β, with a mass of 17 kDa [21]. Interleukin-1β is involved in many physiological processes such as modulation of gene expression and cytokine production, cell adhesion and migration, immune response and, above all, angiogenesis [21]. IL-β and other pro-inflammatory cytokines that activate endothelial cells to produce VEGF-A provide an inflammatory microenvironment for angiogenesis and tumor progression [19]. Studies indicate that the biological pathways and functions regulated by IL-1β and VEGF in the endothelium overlap to some extent [22]. The latest literature also shows that IL-1β induces the secretion of HIF-1α—it stabilizes the hypoxia-induced factor in response to metabolic changes in the cell, i.e., in hypoxic conditions [23]. Elevated levels of IL-1β are observed in many cancers, including colorectal [24], lung [25], breast [26], prostate [27], ovary [28], pancreas [29], and bladder cancer [30]. All three proteins, HIF-1α, ANG-2, and IL-1β, are widely studied in the literature as biomarkers of various cancer diseases. Taking HIF-1α as an example, its levels in hepatocellular carcinoma (HCC) have been studied to determine the effectiveness of transarterial chemoembolization (TACE) treatment. The results indicate that this protein occurs at a level of approximately 1901.62 pg/mL, but after the introduction of TACE treatment this drops to approximately 621.82 pg/mL, measured in the patient’s blood serum using the ELISA test [31]. In the case of lung cancer, an ANG-2 study showed that the mean protein concentration was higher in serum samples of patients (2046.3 ± 1171.3 pg/mL) than in the control group (1269.8 ± 494.1 pg/mL). The analysis was performed using the ELISA test. The authors reported that with the progression of non-small cell lung cancer, the concentration of ANG-2 in blood serum increased, and the concentration was also higher in patients with metastases than in those without [12]. IL-1β, a pro-inflammatory cytokine, is rarely measured in healthy tissues. However, it is reported in the literature that the total daily production of this interleukin is estimated at approximately 6 ng per day, using a specific antibody. However, the measurement of IL-1β in serum enables prediction of the effectiveness of chemotherapy and determination of the patient’s prognosis, and high levels of this protein are associated with the development of aggressive tumor states and poor prognosis [32]. The structure of the IL-1β protein is shown below in Figure 3 [33].

Over the last decades, appropriate methods have continuously been sought for the rapid diagnosis of diseases, including cancer, using plasma or blood serum tests. Such tests are not only non-invasive, but due to the possibility of quite frequent collection of biological material, it is possible to test the levels of compounds after the application of particular treatment methods, which may indicate whether specific therapies contribute to improving the patient’s health. In the field of diagnostics, biosensors that are specific to particular blood biomarkers of various diseases may prove useful. A biosensor is a device that contains a receptor part in its structure, i.e., a given biological element, a transducer, and a signal detector. It enables the conversion of a biological interaction into a measurable analytical signal. The biological component of the receptor layer is selected individually depending on the detected analyte and may include enzymes, tissues, microorganisms, cells, acids, inhibitors, or antibodies [34]. Therefore, the receptor layer’s design depends, as mentioned, on the type of analyte and the concentration and presence of interfering agents. The captured analyte, i.e., the interaction with the receptor layer that generates the signal, can be recorded and visualized using chemical analysis methods. Depending on the type of signal that we want to study, an appropriate method is selected, e.g., electrochemical, optical, thermal, acoustic, or gravimetric [35]. In our work, SPRi biosensors were used, i.e., a device that allows obtaining the concentration of given proteins using a transducer with optical detection, i.e., using the Surface Plasmon Resonance method in the imaging mode (SPRi). Specific antibodies are used as the receptor layer here, which capture analytes from the tested sample in the presence of other matrix components. Then, these antibodies interact with the analyte, which allows its immobilization.

In this work, biosensors based on the detection of Surface Plasmon Resonance in the imaging mode (SPRi) were used. This is a method based on surface plasmons, which are oscillations of free electrons at the boundary of two media with negative and positive real parts of electrical permittivity (metal–dielectric) [36]. The effect of excitation of surface plasmons on the surface of a metal, such as gold, depends on the refractive index of the very thin layers of the medium that is in contact with the metal. Biomolecules can be bonded to the metal surface, and the bonding of subsequent layers leads to changes in the refractive index. These changes can be used to determine, among other things, the thickness of the layers and the concentrations of compounds bound on the surface. The signal generated is directly proportional to the mass immobilized on the metal, and this fact enables quantitative examination of the analyte content in the sample [36]. SPRi devices usually use the Kretschman configuration, illuminating a thin metal layer arranged on a glass prism. This is plane-polarized light at a constant angle, collected by a CCD (charge-coupled device) camera. This allows for the observation of the arranged layer in real time. SPRi devices are increasingly used in medical diagnostics and the study of biomarkers characteristic of diseases due to the excellent sensitivity and efficiency of such biosensors. In addition, they provide clear and resolved images of biological interactions [37].

Another crucial aspect of biosensor technology is the possibility of multiplexing, i.e., testing multiple analytes simultaneously. Here, three receptor layers were made on one biochip, which simultaneously determined three proteins: HIF-1α, ANG-2, and IL-1β. The construction of these three sensors was carried out individually for each of the proteins. After the validation process and appropriate calibration, the receptor layers were placed on one biochip for testing. The standard chip contains nine measurement locations, so each of the receptor layers for each protein were created on three measurement locations, thus filling the entire biochip. An appropriately diluted sample was applied to the three measurement locations with different structures of receptor layers, which allowed for obtaining three results for HIF-1α, ANG-2, and IL-1β, respectively.

## 2. Experimental

### 2.1. Material and Reagents

The following reagents were used for the construction of the biosensor and validation of the analytical method: recombinant HIF-1α protein (R&D Systems, Minneapolis, MN, USA), recombinant human ANG-2 protein (R&D Systems, Minneapolis, MN, USA), recombinant human IL-1β protein (R&D Systems, Minneapolis, MN, USA), recombinant human VEGF-A protein (Abcam, Cambridge, UK), monoclonal mouse antibody against HIF-1α (R&D Systems, Minneapolis, MN, USA), monoclonal mouse antibody against ANG-2 (R&D Systems, Minneapolis, MN, USA), monoclonal mouse antibody against IL-1β (R&D Systems, Minneapolis, MN, USA), monoclonal rabbit antibody against VEGF-A (Abcam, Cambridge, UK), EDC (N-ethyl-N′-(3-dimethylaminopropyl)carbodiimide hydrochloride) (SIGMA, Steinheim, Germany), NHS N-hydroxysuccinimide (Aldrich, Munich, Germany), buffered saline solution (PBS buffer) (Biomed, Lublin, Poland), absolute ethyl alcohol (POCh, Gliwice, Poland), and ethanolamine solution (SIGMA, Steinheim, Germany). The base of the biosensor is a plate with a gold layer (Ssens, Enschede, The Netherlands).

### 2.2. SPRi Apparatus

**Figure 4:**
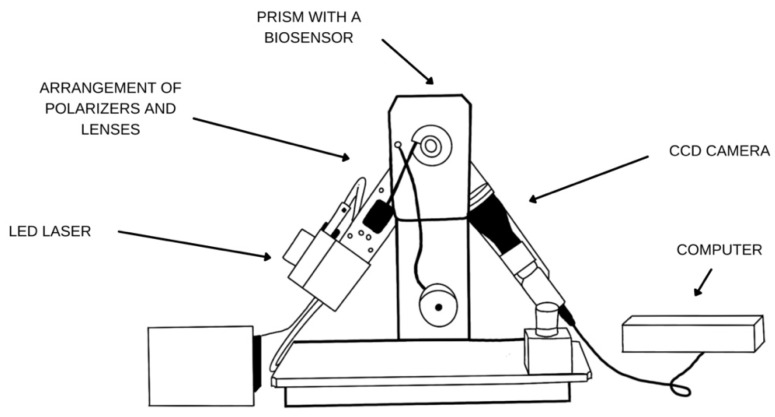
Schematic diagram of the SPRi apparatus with device components. The LED laser emits a beam of light that passes through a system of polarizers and lenses and then hits a prism with a biosensor, a plate with a gold layer. During measurement, the light is collected by a CCD camera and sent to a computer. Own elaboration.

SPRi measurement was performed using a Surface Plasmon Resonance apparatus in the imaging mode, located in the Bioanalysis Laboratory of the Faculty of Chemistry, University of Bialystok. The diagram of the apparatus is shown in Figure 4. This device consists of a diode laser emitting light of wavelength λ = 635 nm, as well as a system of polarizers and lenses through which the emitted light passes. A beam of monochromatic and p-polarized light then passes through a prism with a biosensor located on it, and the reflected light is collected by a CCD camera, where it is converted into an image. The equipment also consists of movable elements, enabling it to operate in an angular range from 30° to 75°. This allows the user to select the appropriate angle, which is determined individually for each biosensor, i.e., for each glass plate with a gold layer on which the appropriate antibody layers are immobilized.

### 2.3. Biological Material

The biological material consisted of three control samples from patients with inflammation of the prostate and 18 samples from patients diagnosed with bladder cancer.

### 2.4. Procedures

#### 2.4.1. Chip Preparation

**Figure 5:**
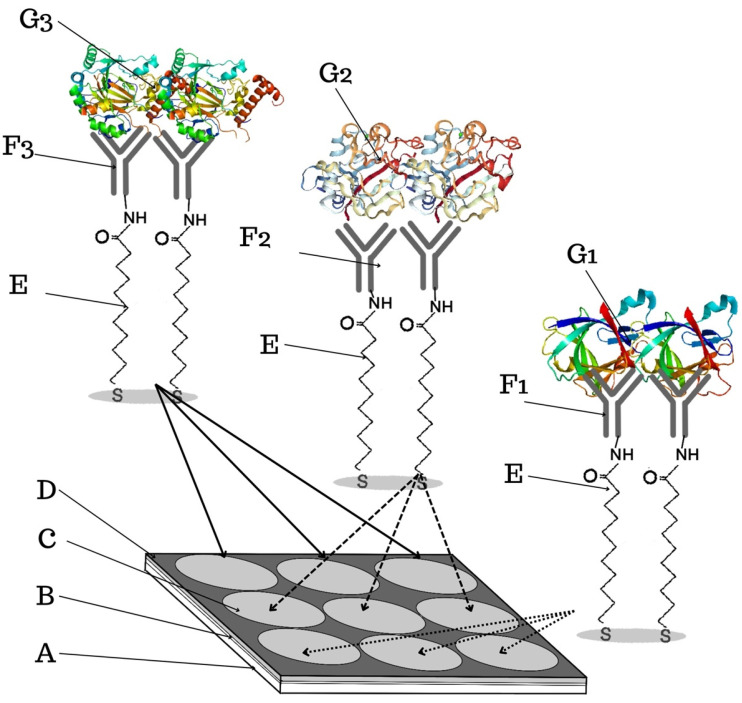
Construction of the biosensor for the determination of HIF-1α, ANG-2, and IL-1β (A—BK7 glass layer; B—titanium layer; C—gold layer; D—polymer foil; E—thiol–mercaptoundecanoic acid 11-MUA; F_1_—IL-1β-specific antibody; F_2_—ANG-2-specific antibody; F_3_—HIF-1α-specific antibody; G_1_—IL-1β protein; G_2_—ANG-2 protein; G_3_—HIF-1α protein). Each generated receptor layer (thiol–antibody) is separate for a given protein. In this way, three measurement spots for detecting each protein were created on one chip, which can be determined simultaneously in one measurement cycle. Own elaboration.

**Figure A1:**
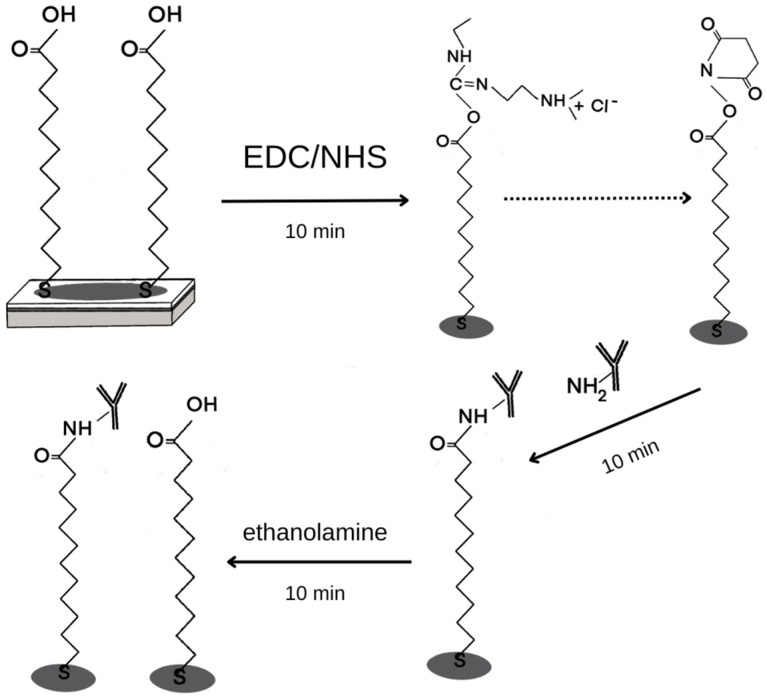
Steps for immobilization of the antibody layer (illustration by the authors). To immobilize the antibody on the gold surface with a thiol layer, a mixture of EDC and NHS is applied in a 1:1 volume ratio. The carboxyl group of the thiol is converted into an ester group by EDC, while NHS modifies the ester into a short-lived NHS-ester. In the next step, the applied antibody forms a covalent amide bond with the NHS-ester. To prevent non-specific adsorption, ethanolamine is applied, which deactivates active NHS-esters and also prevents the attachment of other components to thiol molecules that have not bound to the antibody.

The structure of the biosensor is shown in Figure 5. The chip is made of BK7 glass with sputtered layers of titanium (1 nm) and gold (50 nm). To further immobilize the antibody on the gold plate, the chip is immersed in an alcoholic solution of 11-MUA (11-mercaptoundecanoic acid) thiol, at a concentration of 1 mM, for 24 h. After this time, the chip is washed with ethyl alcohol and dried in a stream of argon. A foil is placed on the prepared chip, which enables the separation of the nine measurement sites. To immobilize a layer of antibody specific for HIF-1α, ANG-2, or IL-1β, a mixture of EDC (0.4 M) and NHS (0.1 M) is applied. EDC converts the carboxyl group of the thiol into an ester group, while NHS modifies the resulting ester into an active, short-lived NHS-ester. After 10 min, the solution is removed from the biosensor surface and the antibody–ligand solution is applied for another 10 min. The NHS-ester forms a covalent amide bond with the amino groups of the antibody. The next step is to apply ethanolamine (1.0 M) to each of the nine active sites of the biosensor. Ethanolamine deactivates active NHS-esters that have not attached to the antibody, and also repels the OH group from the carboxyl group of the thiol so that protein molecules do not attach in the same place—it prevents non-specific adsorption. The immobilization steps are shown in Figure A1 in Appendix A. The chip is then rinsed with PBS buffer and prepared to capture the target analyte from the sample. To be able to mark all three proteins simultaneously, antibodies are immobilized on the surface of one gold chip—on three places on the gold layer, an antibody specific for HIF-1α, on the next three, an antibody specific for ANG-2, and then in the last row on three places, an antibody specific for IL-1β. Chips with 12 gold places, i.e., measurement places, were also used for the studies. These have four places for each of the three analyzed proteins. The division of the chip into individual measurement locations is obtained using a polymer foil, visible in Figure 5. After the determinations are made, the 11-MUA surface can be regenerated. For this purpose, 50 mM NaOH is applied to the active sites of the biosensor for about 5 min, and then the chip is washed with distilled water. This regeneration cycle should be repeated three times, after which the plate is ready to be used for further determinations.

#### 2.4.2. SPRi Measurement

After preparation of an antibody layer specific for HIF-1α, ANG-2, or IL-1β, and appropriate washing of the biosensor using PBS buffer, it was placed on the prism of the SPRi device using oil immersion. The SPRi signal of the receptor layer was measured. A total of 3 µL of samples containing HIF-1α, ANG-2, and IL-1β were applied to the chip and left for 10 min. After washing with PBS buffer, subsequent measurements were taken and saved as images. The analytical signal is the difference in reflected light before and after interaction with the analyte. Image conversion to a quantitative signal was performed using ImageJ software (version 1.53, National Institutes of Health, NIH). The biosensor validation process was performed separately for each protein on three other measurement chips to establish the appropriate concentrations of receptor layers, calibration curves, and proper sample dilutions for each protein. The determinations in natural samples were performed simultaneously for all three proteins on one measurement chip.

## 3. Results and Discussion of Biosensor Formation and Its Validation

### 3.1. Formation of Layers on the Biosensor Surface

**Figure 6:**
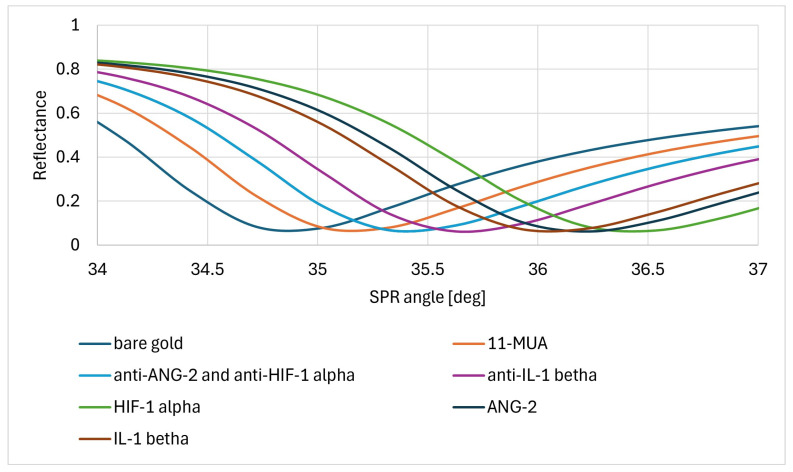
The course of model SPR curves. For each of the distinguished layers present on the biosensor surface, data were collected at angles from 34 to 37 degrees, in 0.1-degree increments. Each of the curves for a given layer is shifted towards larger angle values relative to the curve of the preceding layer.

The formation of successive biosensor layers was confirmed by plotting SPR curves. After the 11-MUA thiol layer was formed, the biosensor was placed using oil immersion on the prism of the SPRi device and the first measurement was performed. EDC, NHS, specific antibodies, and ethanolamine were then applied according to the procedure described in Section 2.4.1. The concentrations of antibodies specific for HIF-1α, ANG-2, and IL-1β were 50.00 ng/mL, 5 μg/mL, and 100 ng/mL, respectively. After immobilization of the ligand–antibody layer, further measurements were performed. Next, solutions containing HIF-1α, ANG-2, and IL-1β, each with a concentration of 100 pg/mL, were applied to the active sites with the appropriate antibody. After waiting for 10 min and washing, further measurements of the protein layer were made. Data acquisition was performed for each layer at angles ranging from 34 to 37 degrees. The SPR curves were adjusted to the experimental data using WinSpall SPR curve modeling software (version 3.02, Res-Tec, Rosenheim, Germany). The SPR curves derived from anti-HIF-1α and anti-ANG-2 antibodies overlap in the figure due to the very similar masses of these two antibodies. Each of the SPR curves is shifted towards higher values of the SPR angle relative to the SPR curve of the preceding layer, which confirms the presence of newly formed layers. The SPR curves are shown in Figure 6 (Model SPR curves).

### 3.2. Optimization of Concentrations of Ligand–Antibody Monolayers

The appropriate concentrations of antibody ligands were determined by applying different concentrations of antibodies specific for HIF-1α, ANG-2, and IL-1β to the active sites of the biosensor. The following concentrations were tested for each compound:

- HIF-1α antibody concentration: 1.00, 5.00, 10.00, 50.00, and 100.00 ng/mL;
- ANG-2 antibody concentration: 1.00, 5.00, 10.00, 15.00, and 20.00 ug/mL;
- IL-1β antibody concentration: 5.00, 10.00, 50.00, 100.00, and 1000.00 ng/mL.

**Figure A2:**
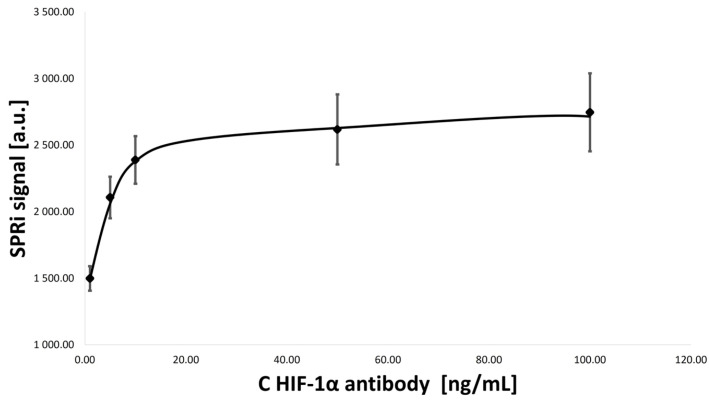
Antibody saturation curve for HIF-1α.

**Figure A3:**
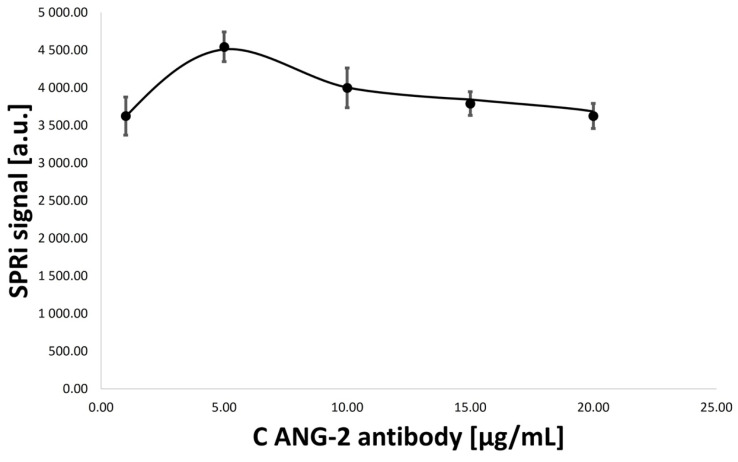
Antibody saturation curve for ANG-2.

**Figure A4:**
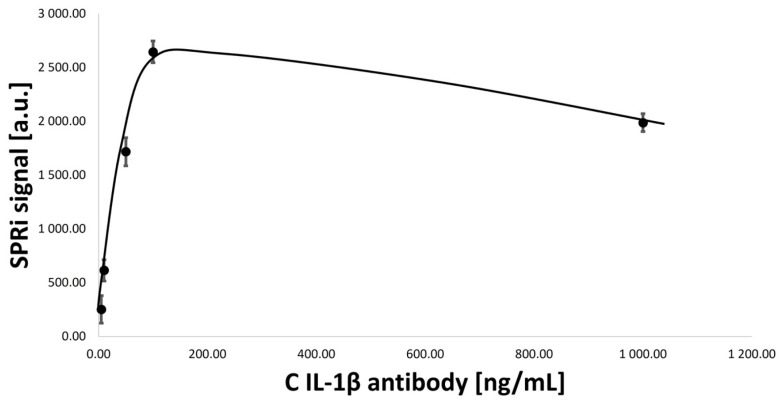
Antibody saturation curve for IL-1β.

The difference in the intensity of reflected light was examined on a layer of bound 11-MUA and then on immobilized antibodies, as described in Section 2.4.1 and Section 2.4.2. The chip with the 11-MUA layer formed was placed on the prism of the SPRi device and the appropriate angle was selected. The first measurements were taken. Then, EDC and NHS were applied, and after 10 min and washing with PBS buffer, the appropriate antibody was used. After another 10 min, excess solutions were removed from the measurement sites, and ethanolamine was applied to avoid non-specific adsorption. In the final step, the ethanolamine was removed, the chip was washed with PBS buffer, and further measurements were taken. The analytical signal is the difference in the intensity of the reflected light before and after applying the appropriate antibody. This procedure was performed for all three antibodies. The SPRi signals and antibody concentration increase up to a certain value, and then a plateau is observed in the saturation curves. The saturation curves are shown in Figure A2, Figure A3 and Figure A4 in Appendix A. Above a concentration of 50.00 ng/mL for HIF-1α, 5.00 μg/mL for ANG-2, and 100.00 ng/mL for IL-1β, no more ligand binding occurs on the biosensor surface. These are the highest concentrations that will enable capture of the analytes of interest from the sample with the greatest accuracy.

### 3.3. Analytical Response to HIF-1α, ANG-2, and IL-1β Concentrations

**Figure 7:**
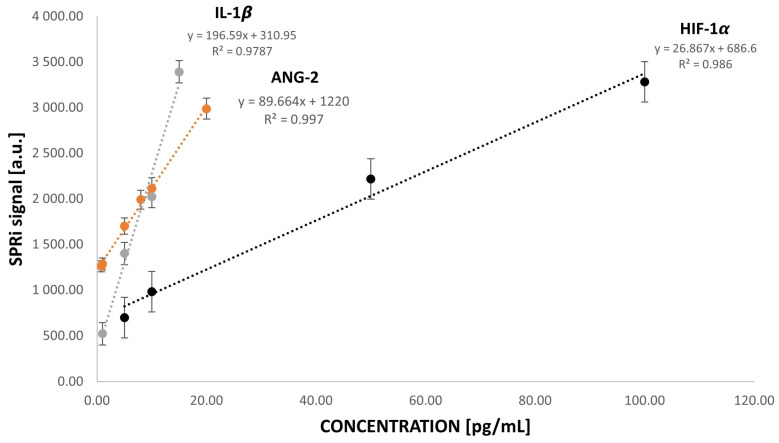
HIF-1α, ANG-2, and IL-1β calibration curves. Ligand/antibody concentration for HIF-1α = 50.00 ng/mL, ligand/antibody concentration for ANG-2 = 5.00 μg/mL, and ligand/antibody concentration for IL-1β = 100.00 ng/mL. Calibration curves were prepared using standard solutions prepared in PBS buffer.

To verify the response of the biosensor, calibration curves were plotted. The SPRi signal was measured in the following concentration ranges: for HIF-1α, from 5.00 to 100.00 pg/mL; for ANG-2, from 1.00 to 20.00 pg/mL; and for IL-1β, from 1.00 to 15.00 pg/mL. Initially, the studies were performed with more comprehensive ranges of protein concentrations: for HIF-1α, from 1 to 1000 pg/mL; for ANG-2, from 1 to 200 pg/mL; and for IL-1β, from 1 to 50 pg/mL. These were selected by analyzing the literature and the levels of these proteins in the plasma of healthy people and of those who have cancer. Measurements were performed and the analytically useful ranges, i.e., the rectilinear part of the calibration curves, were selected from the obtained curves. The experimental procedure is described in Section 2.4.1 and Section 2.4.2. EDC and NHS were applied to the measurement sites with the prepared 11-MUA thiol layer, then the excess solution was removed, and the antibody was applied. In the last step, ethanolamine was also applied. Each cycle lasted 10 min, and the chip was washed with PBS buffer between cycles. After antibody immobilization, the receptor layer was measured. Then, after applying solutions containing the appropriate protein, after 10 min and washing with PBS buffer, further measurements were performed. The results are shown in Figure 7. The limits of detection and quantification were also determined for all three biomarkers. The calculation results are presented in Table 1. The detection limits and quantification limits were determined as described below.

The only preparation of samples conducted before performing the test was their dilution. Appropriate dilution allows the concentration values to be read from the range of calibration curves. The range of curves due to the dilutions used in studying biological samples is sufficient for the tests.

To calculate the limit of detection (LOD) and limit of quantification (LOQ) values, the following formulas were used: LOD = (3.3 × SD)/a and LOQ = (10 × SD)/a, where SD is the standard deviation and a is the slope of the calibration curve.

### 3.4. Precision, Accuracy, and Repeatability of the Biosensor

The precision and accuracy of the determinations made using the SPRi biosensor sensitive to HIF-1α, ANG-2, and IL-1β were determined by threefold measurement of the concentrations of standard solutions with the following actual concentrations: for HIF-1α, 5.00, 10.00, 50.00, and 100.00 pg/mL; for ANG-2, 1.00, 5.00, 10.00, and 20.00 pg/mL; and for IL-1β, 1.00, 5.00, 10.00, and 15.00 pg/mL. The standard deviation (SD), the arithmetic mean of the concentrations from three measurement repetitions (C_mean_), and the coefficient of variation (CV) were then calculated. Low standard deviation values indicate high precision. A CV value lower than 20% indicates high precision of determinations. The results for the three compounds are presented in Table 2 (results of precision and accuracy parameters of protein determinations using the SPRi biosensor).

The repeatability of the biosensor was tested by threefold quantitative analysis of a real sample. Repeatability was determined using the average value of the coefficient of variation (CV). The CV values were 7.18% for HIF-1α, 5.06% for angiopoietin-2, and 2.57% for interleukin-1β. These CV values indicate that the determinations made in a sample of biological material are repeatable.

### 3.5. The Influence of Interferences on the Analytical Signal

A determination in biological material is associated with the presence of many other components in the sample, which may influence the final analytical signal and distort the quantitative result. It is therefore necessary to check whether the design selectively captures the analyte from the samples. As mentioned earlier, all selected compounds take part in the angiogenesis process, and the presence of one of them affects the activity of others. For example, IL-1β induces HIF-1α secretion [23,24,25], and many biological pathways of IL-1β and VEGF-A may overlap [21]. Moreover, in the presence of VEGF, ANG-2 acts as a pro-angiogenic factor, and without its presence, it affects vascular regression. The angiogenic effect of ANG-2 also requires the presence of HIF-1α [13]. Each protein was mixed with solutions of selected interferents in a concentration ratio of 1:10 and 1:100. The selected protein interferents were: for HIF-1α—ANG-2, IL-1β, and VEGF-A; for ANG-2—HIF-1α, IL-1β, and VEGF-A; and for IL-1β—HIF-1α, ANG-2, VEGF-A. The antibodies specific to the detected compound (HIF-1α, ANG-2, or IL-1β) were immobilized on the gold surface according to the procedure (Section 2.4.1). Then, the measurement was performed according to the procedure described in Section 2.4.2. The biosensor is specific to the compound being detected, and so it selectively captures the tested analyte from the sample in the presence of other components found in biological fluids. The parameter of the specificity test is the determined recovery value. The average recovery values obtained for HIF-1α (103.33%), ANG-2 (106.97%), and IL-1β (100.44%) indicate the high specificity of the constructed biosensor.

### 3.6. Determination in Biological Samples

To verify the operation of the biosensor using the developed method, HIF-1α, ANG-2, and IL-1β were simultaneously determined in three plasma samples from patients with inflammation of the prostate (control group) and in 18 plasma samples from patients with diagnosed bladder cancer. The aim was to demonstrate the usefulness of the method for determinations in biological material. An antibody layer was generated on the gold plate according to the EDC/NHS covalent immobilization protocol, and then ethanolamine was applied to avoid non-specific adsorption. Antibodies sensitive to each compound had a specific location on the measurement chip: the first row of measurement locations with an exposed gold layer was intended to analyze HIF-1α levels, the second for ANG-2, and the third for IL-1β, respectively. After SPR analysis of the receptor layers, diluted samples were applied to these locations. One sample was applied to three locations, one from each row. In this way, we could obtain information on the concentration of each protein in a single measurement. After 10 min of washing, further SPR measurements were performed. Plasma samples were appropriately diluted using PBS buffer: 100-fold for HIF-1α, and 10-fold for ANG-2 and IL-1β. This method does not require special preparation of samples, except for appropriate dilution. The dilutions were selected experimentally by analyzing the obtained SPR signals falling within the middle part of the calibration curves. The results obtained for concentrations are presented in Table 3. Study group samples were numbered from 1 to 18, and control samples from 1C to 3C.

The preliminary results obtained indicate that the biosensor is suitable for determining selected pro-angiogenic factors. The average concentration of each of the tested compounds is higher in the study group than in the control group (concentration of HIF-1α: 4.644 ± 2.573 ng/mL vs. 0.805 ± 0.324 ng/mL; concentration of ANG-2: 0.124 ± 0.029 ng/mL vs. 0.036 ± 0.013 ng/mL; concentration of IL-1β: 0.145 ± 0.055 ng/mL vs. 0.024 ± 0.025 ng/mL). To confirm the correctness of the experiment, the results were compared to literature data in which the authors used a certified method, the ELISA test. HIF-1α levels in the blood plasma of patients with hepatocellular carcinoma showed a higher concentration in the patient group (mean concentration 1.092 ng/mL) than in the control group (mean concentration 0.622 ng/mL) [31]. The concentration in the patient group may be reduced due to the use of transarterial chemoembolization (TACE) treatment. Additionally, the study indicated that this protein may be a prognostic factor. Comparing the obtained concentrations with those obtained by our method, there is a significant similarity in HIF-1α levels. ANG-2 was also studied in samples from patients with bladder cancer (median 0.205 ng/mL) and in the control group (median 0.309 ng/mL). Compared to our results, the concentrations are similar, but in the patient group, we obtained higher concentrations than in the control group, which was not present in the results of T. Szarvas’s team. This may be due to the treatment used for the patients from the study team in Germany [38]. In the study by V. Dmytryk’s team, IL-1β levels in control samples did not exceed 0.15 ng/mL; in research samples, the level was higher and reached approximately 0.3 ng/mL. The concentration levels of this protein obtained by the SPRi biosensor method compared to the literature ELISA are similar [39]. The determination and comparison confirmed the correctness of this biosensor’s operation. To obtain information on the usefulness of this biosensor as a biomarker, it is necessary to analyze a more significant number of samples and perform an ELISA test on the same samples. However, the obtained results do not exclude the fact that the biosensor indicates higher HIF-1α, ANG-2, and IL-1β concentrations in samples from patients with bladder cancer compared to the control group.

## 4. Conclusions

A biosensor sensitive to HIF-1α, ANG-2, and IL-1β was constructed for the simultaneous detection of these three proteins in blood plasma samples. Surface Plasmon Resonance imaging (SPRi) was used for the experiments. The method did not require the use of labels, and the measurement procedure for one measurement cycle (12 samples), including the preparation of the antibody–ligand layer, took less than an hour, which is undoubtedly an advantage of this method. A very small amount of sample is used for testing (3 μL), and this method can be applied as three separate analytical methods each for the determination of one selected compound. The validation process was conducted separately for HIF-1α, ANG-2, and IL-1β.

In the first stage, the optimal concentration of ligands—the receptor layer—was determined. For each of the pro-angiogenic factors, a separate curve was plotted, showing the dependence of the SPRi signal on concentration. Determination of the equation of the curve then enabled the determination of concentrations in biological samples. As a result of validation, the LOD and LOQ values of the method were obtained. The results indicate the possibility of determining very low concentrations, of the order of pg/mL (HIF-1α: LOD = 0.09 pg/mL, LOQ = 0.27 pg/mL; ANG-2: LOD = 0.01 pg/mL, LOQ = 0.039 pg/mL; and IL-1β: LOD = 0.010 pg/mL, LOQ = 0.02 pg/mL). Determining other validation parameters indicated that the developed method’s results were highly precise. The biosensor is also specific for the tested proteins.

To verify whether the constructed biosensor can simultaneously determine HIF-1α, ANG-2, and IL-1β in natural samples, testing was performed on plasma from patients with inflammation of the prostate (as a control group) and a study group of patients with diagnosed bladder cancer. Comparing the results for the study and control groups, it is found that the average concentration of each of the tested compounds is higher in the study group than in the control group (C_HIF-1α_: 4.644 ± 2.573 ng/mL vs. 0.805 ± 0.324 ng/mL; C_ANG-2_: 0.124 ± 0.029 ng/mL vs. 0.036 ± 0.013 ng/mL; and C_IL-1β_: 0.145 ± 0.055 ng/mL vs. 0.024 ± 0.025 ng/mL). The concentration levels obtained are similar to those obtained by other research teams using a certified method such as the ELISA test [31,38,39]. The results allow us to formulate a preliminary hypothesis that HIF-1α, ANG-2, and IL-1β may be biomarkers characteristic of bladder cancer, having elevated concentrations in patients with that disease. Confirmation of this will require extensive research on a larger number of samples from patients and control subjects, as well as testing of different types of samples, taking into account the malignancy of cancer lesions, metastases, and treatments already administered. In addition, a broad statistical analysis of the results is needed to verify certain relationships. Nevertheless, the preliminary research presented here confirms the effectiveness and usefulness of the developed SPRi biosensor, which provides accurate and precise results and can also be used in subsequent, broader analyses including the use of other biological samples, such as urine samples.

## Figures and Tables

**Table 1:** Values of detection and quantification limits.

	HIF-1α	ANG-2	IL-1β
LOD [pg/mL]	0.09	0.01	0.01
LOQ [pg/mL]	0.27	0.039	0.02

**Table 2:** Results of precision and accuracy parameters of protein determinations using the SPRi biosensor.

**Concentration of the Standard Solution HIF-1α [pg/mL]**	**SD [pg/mL]**	**C_mean_ [pg/mL]**	**CV [%]**
5.00	0.72	5.14	14.09
10.00	1.91	12.20	8.28
50.00	1.83	44.85	4.08
100.00	6.43	75.04	8.57
**Concentration of the standard solution ANG-2 [pg/mL]**	**SD [pg/mL]**	**C_mean_ [pg/mL]**	**CV [%]**
1.00	0.13	1.93	6.83
5.00	0.18	6.26	2.85
10.00	1.18	12.41	9.51
20.00	1.14	20.08	5.70
**Concentration of the standard solution IL-1β [pg/mL]**	**SD [pg/mL]**	**C_mean_ [pg/mL]**	**CV [%]**
1.00	0.20	1.19	16.94
5.00	0.46	4.73	9.66
10.00	0.91	10.16	8.92
15.00	0.54	15.005	3.62

**Table 3:** Results for concentrations of tested proteins in samples from patients with bladder cancer and in control samples.

Sample Number	Concentration of HIF-1α [ng/mL]	Concentration of ANG-2 [ng/mL]	Concentration of IL-1β [ng/mL]
1	6.667	0.143	0.208
2	0.655	0.134	0.187
3	5.983	0.088	0.190
4	2.321	0.129	0.247
5	5.565	0.129	0.117
6	2.400	0.093	0.084
7	1.442	0.136	0.167
8	6.526	0.144	0.187
9	2.492	0.178	0.165
10	7.910	0.151	0.186
11	4.335	0.080	0.100
12	6.085	0.086	0.163
13	4.973	0.120	0.062
14	2.575	0.118	0.150
15	9.391	0.087	0.092
16	7.885	0.154	0.070
17	1.376	0.155	0.057
18	5.008	0.102	0.184
1C	1.074	0.037	0.053
2C	0.445	0.024	0.014
3C	0.897	0.049	0.006
Mean [ng/mL]	4.644	0.124	0.145
Mean_control_ [ng/mL]	0.805	0.036	0.024
SD [ng/mL]	2.573	0.029	0.055
SD_control_ [ng/mL]	0.324	0.013	0.025

## Data Availability

Dataset available on request from the authors.
